# A Decreased Level of Serum Soluble Klotho Is an Independent Biomarker Associated with Arterial Stiffness in Patients with Chronic Kidney Disease

**DOI:** 10.1371/journal.pone.0056695

**Published:** 2013-02-19

**Authors:** Masashi Kitagawa, Hitoshi Sugiyama, Hiroshi Morinaga, Tatsuyuki Inoue, Keiichi Takiue, Ayu Ogawa, Toshio Yamanari, Yoko Kikumoto, Haruhito Adam Uchida, Shinji Kitamura, Yohei Maeshima, Kazufumi Nakamura, Hiroshi Ito, Hirofumi Makino

**Affiliations:** 1 Department of Medicine and Clinical Science, Okayama University Graduate School of Medicine, Dentistry and Pharmaceutical Sciences, Okayama, Japan; 2 Department of Chronic Kidney Disease and Peritoneal Dialysis, Okayama University Graduate School of Medicine, Dentistry and Pharmaceutical Sciences, Okayama, Japan; 3 Department of Cardiovascular Medicine, Okayama University Graduate School of Medicine, Dentistry and Pharmaceutical Sciences, Okayama, Japan; Baker IDI Heart and Diabetes Institute, Australia

## Abstract

**Background:**

Klotho was originally identified in a mutant mouse strain unable to express the gene that consequently showed shortened life spans. In humans, low serum Klotho levels are related to the prevalence of cardiovascular diseases in community-dwelling adults. However, it is unclear whether the serum Klotho levels are associated with signs of vascular dysfunction such as arterial stiffness, a major determinant of prognosis, in human subjects with chronic kidney disease (CKD).

**Methods:**

We determined the levels of serum soluble Klotho in 114 patients with CKD using ELISA and investigated the relationship between the level of Klotho and markers of CKD-mineral and bone disorder (CKD-MBD) and various types of vascular dysfunction, including flow-mediated dilatation, a marker of endothelial dysfunction, ankle-brachial pulse wave velocity (baPWV), a marker of arterial stiffness, intima-media thickness (IMT), a marker of atherosclerosis, and the aortic calcification index (ACI), a marker of vascular calcification.

**Results:**

The serum Klotho level significantly correlated with the 1,25-dihydroxyvitamin D level and inversely correlated with the parathyroid hormone level and the fractional excretion of phosphate. There were significant decreases in serum Klotho in patients with arterial stiffness defined as baPWV≥1400 cm/sec, atherosclerosis defined as maximum IMT≥1.1 mm and vascular calcification scores of ACI>0%. The serum Klotho level was a significant determinant of arterial stiffness, but not endothelial dysfunction, atherosclerosis or vascular calcification, in the multivariate analysis in either metabolic model, the CKD model or the CKD-MBD model. The adjusted odds ratio of serum Klotho for the baPWV was 0.60 (p = 0.0075).

**Conclusions:**

Decreases in the serum soluble Klotho levels are independently associated with signs of vascular dysfunction such as arterial stiffness in patients with CKD. Further research exploring whether therapeutic approaches to maintain or elevate the Klotho level could improve arterial stiffness in CKD patients is warranted.

## Introduction

Chronic kidney disease (CKD) may fundamentally underlie the development of cardiovascular disease (CVD) and appears to be a risk factor for CVD [1]. Patients with CKD are more likely to die of CVD than to develop end-stage renal failure [2]. CKD leads to increased levels of parathyroid hormone (PTH) and fibroblast growth factor 23 (FGF23) and decreased levels of circulating 1,25-dihydroxyvitamin D (1,25D) along with hypocalcemia, hyperphosphatemia, bone disease, vascular calcification and cardiovascular morbidities collectively referred to as chronic kidney disease-mineral and bone disorder (CKD-MBD) [3], [4], [5]. Recent reports suggest that increased levels of FGF23 are a common manifestation of CKD that develop earlier than increased levels of phosphate or PTH [6]. Additionally, the circulating FGF23 level is independently correlated with endothelial dysfunction, possibly due to asymmetrical dimethyl arginine, an endogenous inhibitor of nitric oxide synthase [7].

The Klotho gene, identified as an ‘aging suppressor’ gene in mice, encodes a single-pass transmembrane protein that is predominantly expressed in the distal tubular epithelial cells of the kidneys, parathyroid glands and choroid plexus of the brain [8], [9], [10], [11]. Klotho was originally identified in a mutant mouse strain that could not express the gene, which developed multiple disorders resembling human aging and had a shortened life span [10]. The aging phenotypes include atherosclerosis, endothelial dysfunction, low bone mineral density, sarcopenia, skin atrophy and impaired cognition. In an atherosclerotic mouse model, the *in vivo* gene delivery of Klotho protects against endothelial dysfunction [12]. HMG-CoA reductase inhibition enhances the Klotho protein expression in the kidneys and inhibits atherosclerosis in rats with chronic blockade of nitric oxide synthase [13]. Emerging evidence suggests that a deficiency of Klotho is an early biomarker for CKD [14], [15], [16], [17] and acute kidney injury [18]. There are two forms of Klotho, a membrane form and a secreted form, and each has distinct functions. Membrane Klotho acts as an obligate co-receptor for FGF23, a bone-derived hormone that induces phosphate excretion into the urine [19]. Secreted Klotho is involved in the regulation of nitric oxide production in the endothelium [20], [21], maintenance of endothelial integrity and permeability [22], calcium homeostasis in the kidneys [23] and inhibition of intracellular insulin and insulin-like growth factor-1 signaling [24]. Secreted Klotho proteins are present in human sera and cerebrospinal fluid, suggesting that post-translational cleavage results in the release of Klotho proteins from the cell membrane [25]. The extracellular domain of Klotho is clipped by the membrane-anchored proteases ADAM10 and ADAM17 in order to generate the secreted form [26].

Recently, a sensitive and specific assay was developed for the measurement of soluble Klotho in humans [27]. Low serum Klotho levels have been reported to be associated with poor skeletal muscle strength [28] and the prevalence of CVD [29] and all-cause mortality [30] in community-dwelling adults. The expression of local vascular Klotho has been observed to decrease in human arteries in patients with CKD compared to healthy individuals [31]. Low serum Klotho levels have been reported in patients with diabetes mellitus [32]. However, whether the serum Klotho levels are closely related to signs of vascular dysfunction such as arterial stiffness in patients with CKD is largely unknown. We hypothesized that low serum Klotho levels are associated with signs of vascular dysfunction such as arterial stiffness in patients with CKD. To address this hypothesis, we measured the serum Klotho levels and extensively investigated the relationship between the serum Klotho level and signs of vascular dysfunction, including endothelial dysfunction, arterial stiffness, atherosclerosis and vascular calcification, in CKD patients. The data presented here suggest that a decrease in the serum soluble Klotho level is an independent biomarker of pronounced arterial stiffness in patients with CKD.

## Results

### Patient characteristics

The baseline characteristics of the study population are shown in **Table 1**. A total of 114 CKD patients with a median age of 58 (47–66) years were included in the study. The background causes of CKD included 54 cases of glomerulonephritis (47%), 27 cases of nephrosclerosis (24%), 13 cases of diabetic nephropathy (11%) and 20 cases of “other” (18%). A total of 83 patients were on antihypertensive therapy (71 patients were being treated with angiotensin receptor blockers (ARBs) or angiotensin converting enzyme inhibitors (ACEIs), 58 with calcium channel antagonists and 14 with other agents). Antihyperlipidemic agents were administered to 35 patients and antidiabetic agents were administered to 16 patients. The median serum Klotho level was 616.3 pg/mL, with an interquartile range of 460.0 to 755.5 pg/mL, the value of which was comparable to that reported in a previous study of CKD patients [17] and was higher than that in hemodialysis patients [33], [34].

**Table 1:** Baseline characteristics of the study subjects.

	CKD patients (n = 114)
Age (year)	58 (47–66)
Male sex, n (%)	72 (59%)
Cause of CKD, n	
Glomerulonephritis	54 (47%)
Nephrosclerosis	27 (24%)
Diabetic nephropathy	13 (11%)
Others	20 (18%)
Current medication, n	
ARBs/ACEIs	71 (62%)
CCBs	58 (51%)
MBP (mmHg)	94±13
Serum calcium (mg/dL)	9.3 (9.0–9.5)
Serum phosphate (mg/dL)	3.6±0.7
FECa (%)	0.42 (0.26–0.70)
FEPi (%)	16.0 (11.2–29.6)
Intact PTH (pg/mL)	50 (38–84)
25D (ng/mL)	15 (12–23)
1,25D (pg/mL)	35 (24–50)
FGF23 (pg/mL)	44.4 (31.6–90.0)
Serum Klotho (pg/mL)	616.3 (459.9–755.5)
LDL-cholesterol (mg/dL)	119±34
HDL-cholesterol (mg/dL)	51 (41–64)
Triglycerides (mg/dL)	133 (89–183)
HbA1c (NGSP) (%)	5.7 (5.5–6.0)
CRP (mg/dL)	0.05 (0.02–0.15)
eGFR (ml/min/1.73 m^2^)	48±29
Albuminuria (mg/day)	439 (128–1381)
Serum albumin (g/dL)	3.9 (3.6–4.2)
Hemoglobin (g/dL)	12.6±2.2
Uric acid (mg/dL)	6.9±1.6
FMD (%)	4.7 (3.1–7.6)
baPWV (cm/sec)	1560 (1331–1796)
Max IMT (mm)	0.85 (0.68–1.10)
ACI (%)	4.2 (0–16.4)

ACEI, angiotensin converting enzyme inhibitor; ACI, abdominal aortic calcification index; ARB, angiotensin receptor blocker; baPWV, brachial-ankle pulse wave velocity; CRP, C-reactive protein; 1,25D, 1,25-dihydroxyvitamin D; 25D, 25-hydroxyvitamin D; eGFR, estimated glomerular filtration rate; FECa, fractional excretion of calcium; FEPi, fractional excretion of phosphate; FGF23, fibroblast growth factor 23; FMD, flow-mediated dilatation, HDL, high density lipoprotein; IMT, intima-media thickness; LDL, low density lipoprotein; MBP, mean blood pressure; NGSP, national glycohemoglobin standardization program.

### Relationship between the serum Klotho level and age, renal function, CKD-related mineral metabolism and markers of vascular dysfunction

**Figure 1:**
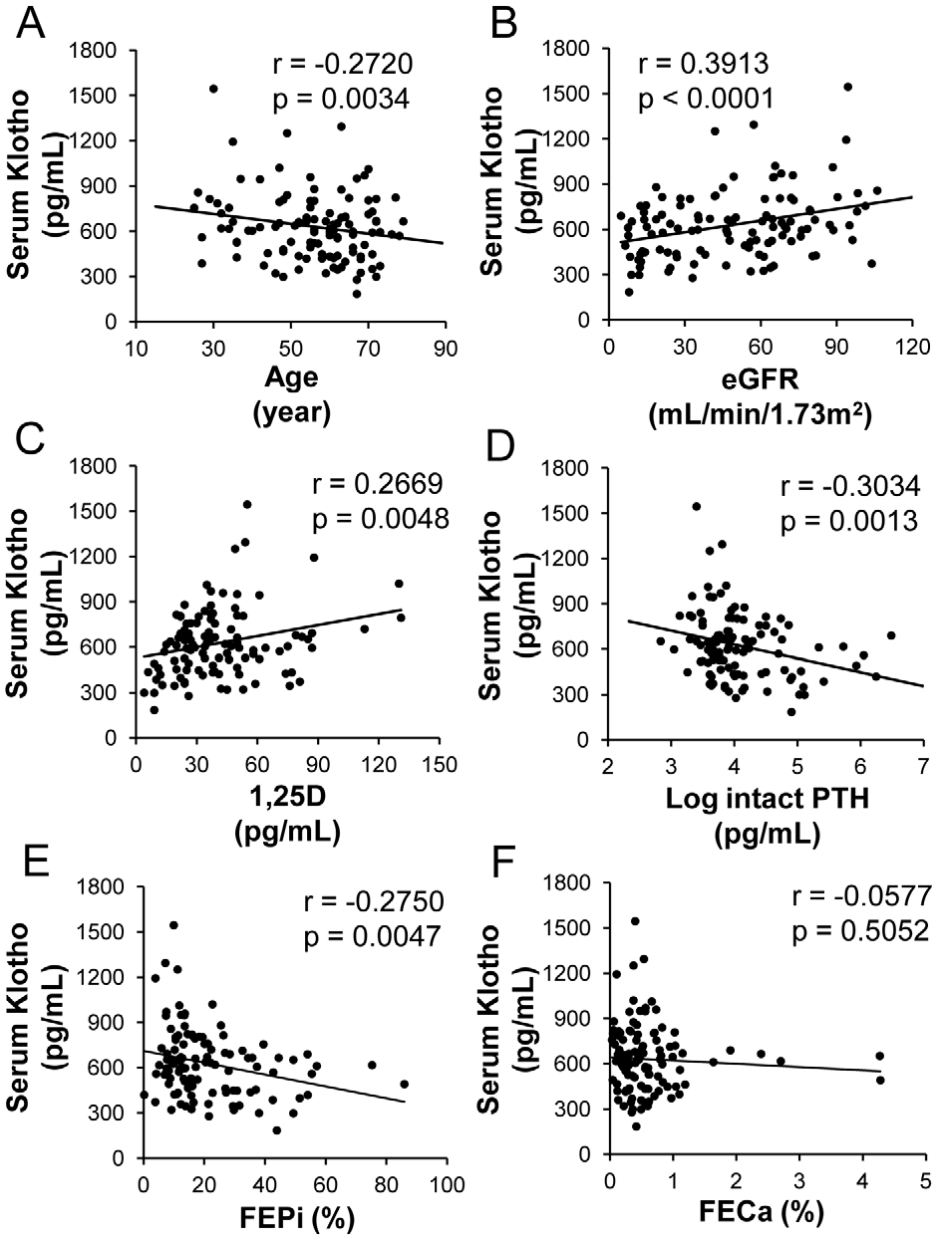
Correlation between the serum Klotho levels (pg/mL) and various parameters. The relationships between the serum Klotho levels and patient age (years) (A), estimated glomerular filtration rate (eGFR) (mL/min/1.73 m^2^) (B) and markers of chronic kidney disease-mineral and bone disorder (CKD-MBD), including 1,25-dihydroxyvitamin D (1,25D) (pg/mL) (C), log intact parathyroid hormone (PTH) (pg/mL) (D), fractional excretion of phosphate (FEPi) (%) (E) and fractional excretion of calcium (FECa) (%) (F) are shown. The serum Klotho levels were inversely correlated with age and positively correlated with eGFR (**A, B**). Regarding CKD-MBD markers, the serum Klotho levels were significantly correlated with 1,25D and negatively correlated with log intact PTH and FEPi; however, no significant correlation was observed with FECa (**C–F**). (**A–F**) N = 114.

Age-dependent changes were recognized in the serum Klotho levels in patients with CKD (**Figure 1A**), as has been reported in healthy subjects [27]. The serum Klotho level was significantly correlated with the eGFR (**Figure 1B**) and decreased along with CKD stages (**Figure S1A**). With regard to markers of CKD-MBD, the serum Klotho level was positively correlated with the 1,25-dihydroxyvitamin D (1,25D) level (**Figure 1C**) and negatively correlated with the log intact parathyroid hormone (PTH) and fractional excretion of phosphate (FEPi) (**Figure 1D, 1E**). The FEPi significantly increased along with declines in the eGFR (univariate regression, r = −0.7228, p<0.0001). There were no correlations between the level of serum Klotho and the fractional excretion of calcium (FECa) (**Figure 1F**) or the 25-hydroxyvitamin D (25D) level (**Figure S2C**). However, correlations were observed between the level of serum Klotho and the level of serum calcium (r = 0.1618; p = 0.0855), the level of serum phosphate (r = −0.1454; p = 0.1426) and log intact FGF23 (r = −0.1751; p = 0.0624) (**Figure S2A, Figure S2B** and **Figure S2D**, respectively).

**Figure 2:**
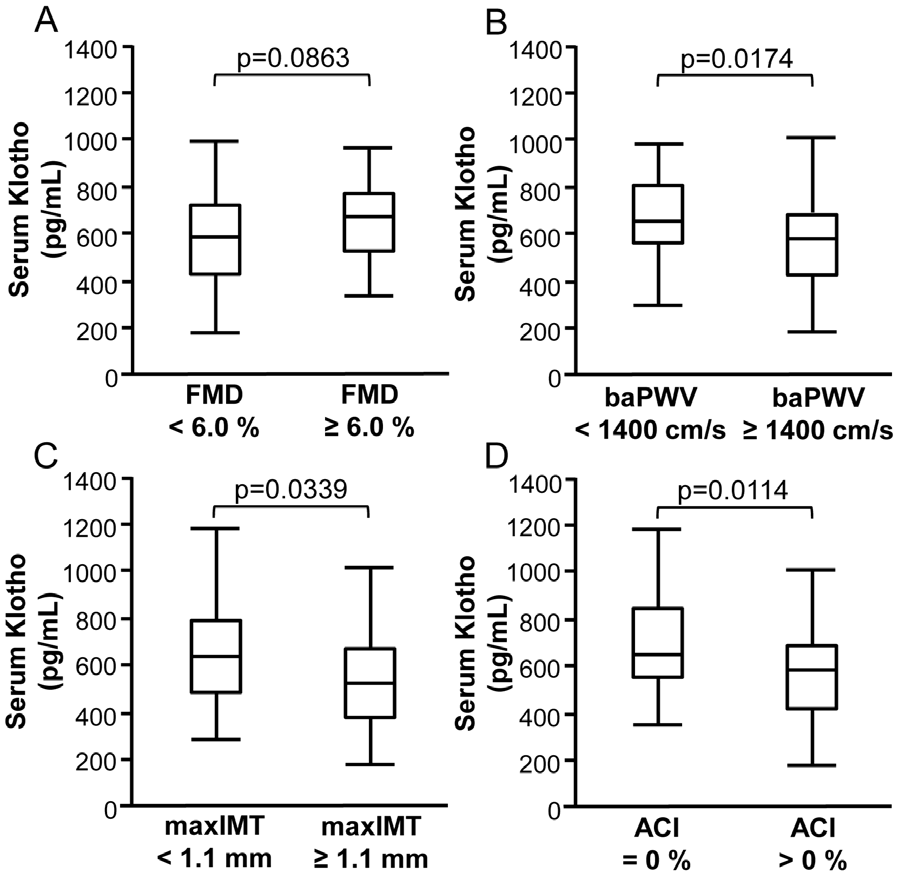
Box and line plots showing the levels of serum Klotho (pg/mL) according to the stratified levels of vascular dysfunction. They include flow-mediated dilatation (FMD) (%), a marker of endothelial dysfunction (A), ankle-brachial pulse wave velocity (baPWV) (cm/sec), a marker of arterial stiffness (B), maximum intima-media thickness (max IMT) (mm), a marker of atherosclerosis (C), and the aortic calcification index (ACI) (%), a marker of vascular calcification (D). The serum Klotho levels were significantly lower in patients with FMD<6.0%, PWV≥1400 cm/s, max IMT≥1.1 mm and ACI>0% compared to patients with FMD≥6.0%, PWV<1400 cm/s, max IMT<1.1 mm and ACI = 0%, respectively (**A–D**). (**A**) N = 70 and n = 40 in FMD<6.0% and FMD≥6.0%, respectively. (**B**) N = 60 and n = 45 in PWV<1400 cm/s and PWV≥1400 cm/s, respectively. (**C**) N = 82 and n = 29 in max IMT<1.1 mm and max IMT≥1.1 mm, respectively. (**D**) N = 28 and n = 75 in ACI = 0% and ACI>0%, respectively. The boxes denote the medians and 25th and 75th percentiles. The lines mark the 5th and 95th percentiles.

We next investigated the association between the serum Klotho level and various markers of vascular dysfunction, including flow-mediated dilatation (FMD), a marker of nitric oxide-dependent endothelial function, brachial-ankle pulse wave velocity (baPWV), a marker of arterial stiffness, maximum intima-media thickness (max IMT), a marker of atherosclerosis, and the abdominal aortic calcification index (ACI), a marker of vascular calcification (**Figure 2**). The serum Klotho levels tended to be lower in patients with FMD<6.0% compared to those with FMD≥6.0% (p = 0.0863) (**Figure 2A**). The serum Klotho levels were significantly lower in patients with PWV≥1400 cm/s, max IMT≥1.1 mm and ACI>0% compared to those with PWV<1400 cm/s, max IMT<1.1 mm and ACI = 0%, respectively (**Figure 2B–D**).

### A multivariate analysis of the determinants of signs of vascular dysfunction, including arterial stiffness, in CKD patients

**Figure 3:**
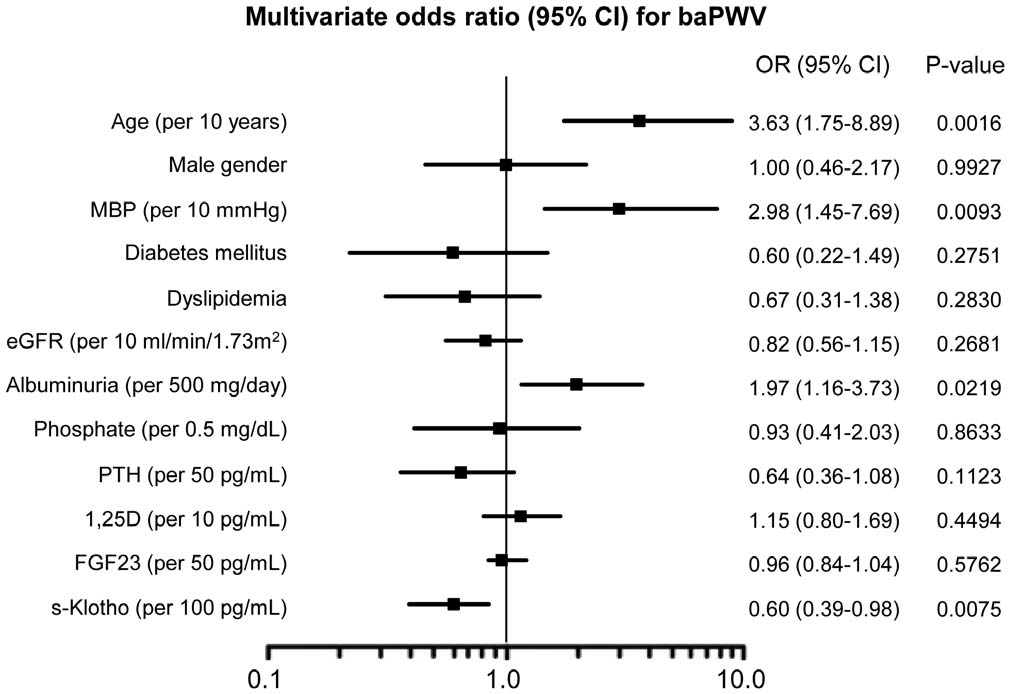
Multivariate odds ratio for ankle-brachial pulse wave velocity (baPWV) among patients with CKD displayed as the odds ratio (OR) (solid boxes) with 95% confidence intervals (CIs) (horizontal limit lines). For continuous variables, the unit of change is given in parenthesis based on the multivariate model described in Table 2. MBP, mean blood pressure; eGFR, estimated glomerular filtration rate; PTH, parathyroid hormone; 1,25D, 1,25-dihydroxyvitamin D; FGF23, fibroblast growth factor 23.

Separate multiple logistic regression models for markers of various signs of vascular dysfunction were analyzed (**Table 2** **and Table S1, S2, S3**). After adjusting for age, gender, mean blood pressure, use of antihypertensive drugs, drinking and current smoking, the serum Klotho level was found to be a significantly independent predictor of baPWV≥1400 cm/sec in a metabolic model that included non-HDL cholesterol, use of antihyperlipidemic agents, hemoglobin A1c and use of antidiabetic agents as other parameters (**Table 2** **, upper panel**). The serum Klotho level was also found to be a significantly independent predictor of baPWV≥1400 cm/sec in a CKD model that included eGFR, albuminuria and hemoglobin as other parameters (**Table 2** **, middle panel**) and a CKD-MBD model that included serum calcium, phosphate, intact PTH, 1,25D and FGF23 as other parameters (**Table 2** **, lower panel**). We performed the same analysis using multiple logistic regression models of the serum Klotho level as a predictor of FMD≥6.0%, max IMT≥1.1 mm and ACI>0%; however, the serum Klotho level was not found to be a significant predictor of any of these parameters (**Table S1, S2, S3**, respectively). Next, a multivariable logistic regression analysis was performed to evaluate the impact of serum Klotho on arterial stiffness assessed by baPWV in CKD patients. This model includes candidate predictors that were selected based on Table 2. The factors significantly associated with baPWV were age, MBP, albuminuria and serum Klotho. The adjusted odds ratios (ORs) for serum Klotho (per 100 pg/mL increase) and albuminuria (per 500 mg/day increase) were 0.60 (95% CI: 0.39 to 0.98; p = 0.0075) and 1.97 (95% CI: 1.16 to 3.73; p = 0.0219), respectively (**Figure 3**).

**Table 2:** A multiple logistic regression analysis of predictors of PWV≥1400 cm/sec.

	β	p
Metabolic model		
serum Klotho	−0.00404	0.0315
non HDL	0.00226	0.8185
antihyperlipidemic drugs	0.42663	0.2660
HbA1c (NGSP)	0.43333	0.4369
antidiabetic drugs	0.43224	0.5107
CKD model		
serum Klotho	−0.00349	0.0431
eGFR	0.01367	0.3911
albuminuria	0.00062	0.1904
Hemoglobin	−0.01483	0.9467
CKD-MBD model		
serum Klotho	−0.00431	0.0368
serum calcium	−0.96331	0.4039
serum phosphate	−0.65510	0.4178
intact PTH	−0.00625	0.2903
1,25D	0.00367	0.8244
FGF23	−0.00052	0.6933

Adjusted for age, gender, mean blood pressure, antihypertensive drug use, drinking and current smoking. CKD, chronic kidney disease; 1,25D, 1,25-dihydroxyvitamin D; eGFR, estimated glomerular filtration rate; FGF23, fibroblast growth factor 23; HDL, high density lipoprotein; MBD, mineral and bone disorder; NGSP, national glycohemoglobin standardization program.

## Discussion

In this study, we measured the serum Klotho levels and determined the relationships between the serum Klotho level and markers of CKD-MBD and vascular dysfunction, including FMD, baPWV, max IMT and ACI in patients with CKD. We herein provide the first evidence in CKD patients that: 1) the serum soluble Klotho level is significantly correlated with markers of CKD-MBD, including the levels of PTH, 1,25D and FEPi; 2) decreased levels of serum Klotho are significantly associated with signs of vascular dysfunction such as pronounced arterial stiffness evaluated by baPWV; and 3) in a multivariate analysis, the serum Klotho level was found to be an independent determinant of marked arterial stiffness, which has been reported to be associated with increased cardiovascular mortality and morbidity.

In this study, the group with lower levels of serum Klotho exhibited significantly lower eGFR levels, as previously reported in CKD patients [17] and patients on hemodialysis [34]. It has been reported that the mRNA and protein expression levels of Klotho are severely reduced in the kidneys of patients with chronic renal failure compared to control subjects [35]. However, it seems that the serum Klotho levels are not completely depleted, even in patients with stage 5 CKD on hemodialysis [34]. This finding suggests that a basal level of Klotho production from other organs than the kidneys, such as the brain and parathyroid glands, might exist in humans, as has been previously reported in mice [8], [9], [10]. A recent study indicated that the transcriptional suppression of Klotho by a protein-bound uremic toxin, indoxyl sulfate, results from CpG hypermethylation of the Klotho gene [36]. Since indoxyl sulfate may play a significant role in the vascular disease and higher mortality observed in CKD patients [37], epigenetic modification of the Klotho gene by a uremic toxin such as indoxyl sulfate might be a mechanism underlying the association between the decline of serum Klotho levels and arterial stiffness in CKD patients observed in the current study.

With regard to markers of CKD-MBD, the serum Klotho level was inversely correlated with the FEPi and log intact PTH and positively correlated with the 1,25D level. The FEPi significantly increased along with declines in the eGFR in the CKD patients evaluated in this study and also in the Chronic Renal Insufficiency Cohort (CRIC) study [6]. The serum Klotho is unable to function as a decoy receptor for FGF23, because Klotho alone does not bind to FGF23 with high affinity. Unlike membrane Klotho, serum Klotho cannot efficiently support FGF23-induced activation of FGF signaling [38]. Instead, serum Klotho may inhibit Type 2a Na-phosphate co-transporter (Npt2a) by decreasing the number of cell-surface Npt2a, thereby reducing cellular phosphate uptake in renal proximal tubular cells [39]. The level of serum Klotho might therefore reflect increased phosphate excretion from the kidneys, which is one of the characteristics of disordered mineral metabolism observed in CKD patients.

To date, several markers have been utilized to assess cardiovascular dysfunction in CKD patients, including FMD, baPWV, IMT and ACI [40], [41], [42], [43], [44]. In the current study, we demonstrated that the level of serum Klotho is an independent determinant of arterial stiffness only defined as baPWV≥1400 cm/s, even after adjusting for age, gender, mean blood pressure, use of antihypertensive drugs, drinking and smoking. In addition, serum Klotho was also a significant predictor of arterial stiffness in the full model including confounders such as age, MBP, diabetes mellitus, dyslipidemia, eGFR, albuminuria, phosphate, PTH, 1,25D and FGF23, and the adjusted odds ratio (OR) for serum Klotho (per 100 pg/mL increase) was 0.60 (95% CI: 0.39 to 0.98; p = 0.0075). There have been some reports discussing the associations between baPWV and CKD-MBD parameters such as phosphate [45], 1,25D [46], PTH [47], [48] and FGF23 [49], [50]; however, these associations are inconsistent. Several reports have shown that increases in aortic stiffness begin as early as CKD stage 2 and increase with the progression to stages 3 and 4 [51], [52]. Conversely, improvements in aortic stiffness have been associated with improved prognoses in patients with end-stage renal disease [53]. The role of serum Klotho in the progression of arterial stiffness has not yet been elucidated in human CKD; however, *in vivo* gene delivery of Klotho into skeletal muscle prevents medial hypertrophy of the aorta in an animal model of atherosclerotic disease [12]. It also improves endothelium-dependent relaxation of the aorta in response to acetylcholine in association with increases in nitric oxide production, suggesting that soluble Klotho plays a protective role against the development of vascular endothelial dysfunction. Although the receptor for soluble Klotho located in the vascular endothelium has not been identified, soluble Klotho regulates calcium influx to maintain the integrity of vascular endothelial cells in a mouse model and in *in vitro* endothelial cell culture studies [22]. The ‘local’ vascular Klotho in human arteries may act as an endogenous inhibitor of vascular calcification and as a cofactor required for vascular FGF23 signaling [31]. Conducting further studies will therefore be necessary in order to investigate how ‘systemic’ serum Klotho interacts with the mechanisms of arterial stiffness in human CKD.

An association between Klotho deficiency and vascular calcification has been reported in aging mice and in a mouse model of CKD [10], [16], [24]. In the assessment of vascular calcification conducted in the current study, the levels of serum Klotho were decreased in CKD patients with ACI>0% compared to those in patients without aortic calcification (Figure 2D), although the levels of serum Klotho were not significantly correlated with the degree of ACI (Figure S2H) or were not independent determinants of ACI (Table S3). There are two possible reasons why the serum Klotho levels are not significantly correlated with the degree of aortic calcification in human CKD patients. First, soft tissue calcification in human CKD may progress more slowly than that observed in murine CKD [16], despite phosphorus and calcium playing major roles in the calcification process in CKD patients. The CKD cohort in our study comprised mostly patients with CKD of stages 1 to 3 (68.4%), which are the early to middle stages of CKD, rather than patients with severe renal dysfunction or uremia that may induce a more procalcific CKD phenotype [54].

Increased serum phosphorus levels are associated with cardiovascular disease in both patients with chronic kidney disease (CKD) and in the general population. High phosphate levels may play a direct role in vascular dysfunction. In the current study, however, there were no significant correlations between the serum phosphate levels and the FMD (r = −0.0530, p = 0.5596), baPWV (r = 0.1217, p = 0.2778), max IMT (r = 0.1030, p = 0.2695) or ACI (r = 0.0245, p = 0.7988). Kestenbaum et al. reported a significant increase in the mortality risk in patients with CKD with phosphate levels higher than 3.5 mg/dL [55]. In our cohort, only 41.4% (46 out of 114) patients exhibited serum phosphate levels higher than 3.5 mg/dL, so the phosphate levels might not correlate with the vascular dysfunction in this study. A recent report demonstrated that a high phosphate level directly affects endothelial dysfunction [56]. Indeed, our data suggest that there is some relationship between the FEPi and FMD (r = −0.2520, p = 0.0077), although the correlation was not statistically significant. Another report using an animal model indicated that changes in extracellular phosphorus concentrations may directly modulate the vascular smooth muscle function [57]. Based on these findings, phosphate could still be a major direct player in the pathogenesis of the vascular dysfunctions observed in patients with CKD.

Membrane Klotho functions as a co-receptor for FGF23, a bone-derived hormone that induces phosphate excretion into the urine [19]. The presence of membrane Klotho determines the target organs of FGF23 and its signaling since most tissues express receptors for FGF. Nakano et al. recently reported that the serum intact FGF23 level is the earliest indicator among various CKD-MBD-related factors and that a high intact FGF23 level and a low 25-hydroxyvitamin D (25D) level independently predict poor renal outcomes, even after adjusting for other MBD-related factors, in patients with pre-dialysis CKD [58]. However, the serum Klotho level was not evaluated in that report, and the exact functions of serum soluble Klotho have yet to be defined [59]. Therefore, whether an excess level of FGF23 and the occurrence of adverse outcomes in patients with CKD are mediated by a deficiency of serum Klotho remains unclear [60].

There have been discrepancies among the study results concerning the correlation between the serum Klotho levels and GFR in patients with CKD [17], [32], [61]. One study found that the plasma Klotho level was not related to the kidney function in patients with CKD, but this study population included nearly 40% patients with diabetes mellitus (39.4%) [61]. In contrast, the serum levels of soluble Klotho were decreased in patients with early stages of CKD in a different study including 15.4% diabetes mellitus cases [17]. These discrepancies may be due to two possible causes. First, including diabetic patients in the CKD cohort may underestimate the level of serum Klotho, since the level of serum Klotho is lower in diabetic patients compared to non-diabetic patients [32]. Second, several ELISA kits to detect the level of soluble Klotho are commercially available, potentially leading to different results in terms of the association of serum Klotho with the renal function.

Our study has several limitations and strengths that should be kept in mind when interpreting the results. First, the cross-sectional nature of our observations precluded making any cause-effect inferences about the relationship between the serum Klotho level and arterial stiffness in CKD patients. Second, we lacked data regarding the patients' dietary phosphorus intake, a critical factor for CKD-MBD and the CKD-associated incidence of CVD, which may be related to the serum Klotho level. However, this weakness is, in part, offset by the criteria of our study because patients who were being treated with vitamin D or phosphate binders were excluded.

In conclusion, the serum Klotho level was found to significantly correlate with markers of CKD-MBD and is an independent biomarker of arterial stiffness in patients with CKD. Further studies are required to elucidate which intervention(s) can modulate the level of serum Klotho, as has been reported in rodents [13], [62], [63], and whether any interventions to increase or maintain the serum Klotho level can prevent cardiovascular events and mortality in CKD patients.

## Subjects and Methods

### Subjects

The subjects in this study were patients admitted to the Renal Unit of Okayama University Hospital. All patients were diagnosed with CKD according to their estimated glomerular filtration rate (eGFR) and the presence of kidney injury as defined by the National Kidney Foundation K/DOQI Guidelines [64], [65]. Hypertension was defined as systolic blood pressure (SBP)≥140 mmHg or diastolic blood pressure (DBP)≥90 mmHg or the use of antihypertensive drugs. The eGFR was calculated according to the simplified version of the Modification of Diet in Renal Disease (MDRD) formula [eGFR = 194×(sCr)^−1.094^×(age)^−0.287^(if female×0.739)] [66]. Smoking status (current smoker vs. non-smoker) was determined from a medical interview. Current drinking was defined as drinking alcohol at least two times per week in the last year. All procedures in the present study were carried out in accordance with institutional and national ethical guidelines for human studies, and guidelines proposed in the Declaration of Helsinki. The ethics committee of Okayama University Graduate School of Medicine, Dentistry and Pharmaceutical Sciences approved the study. Written informed consent was obtained from each subject. This study was registered with the Clinical Trial Registry of the University Hospital Medical Information Network (registration number UMIN000003614). According to the established protocol, we excluded any patients with established atherosclerotic complications (coronary artery disease, congestive heart failure or peripheral vascular disease). Patients with nephrotic syndrome and patients who were being treated with vitamin D or phosphate binders were excluded. None of the patients had an acute infection at the time of the study.

### Laboratory measurements

Each subject's arterial blood pressure was measured by a physician after a 10 minute resting period to obtain the systolic and diastolic pressures. The mean blood pressure (MBP) was calculated as DBP+(SBP−DBP)/3. All samples were obtained from patients in the morning after 12 hours of fasting. The soluble α-Klotho (Klotho) concentrations in the serum were measured using an ELISA system (Immuno-Biological Laboratories, Gunma, Japan) [27]. The serum levels of intact FGF23 were determined using a commercial sandwich ELISA kit (Kainos Laboratories, Inc., Tokyo, Japan). The serum levels of total protein, albumin, creatinine, calcium, inorganic phosphate and glucose, as well as the urinary levels of albumin, creatinine, calcium and inorganic phosphate, were measured in all patients. The serum levels of 1,25-dihydroxyvitamin D (1,25D) and 25-hydroxyvitamin D (25D) were measured using a radioimmunoassay and the serum intact PTH levels were measured using an immunoradiometric assay. The fractional excretion of phosphorus (FEPi) and calcium (FECa) were calculated as (urine mineral×serum creatinine)/(serum mineral×urine creatinine).

### Vascular assessments

#### Endothelial dysfunction

Flow-mediated dilatation (FMD) and endothelium-independent vasodilatation (nitroglycerin-mediated dilatation; NMD) of the brachial artery were assessed noninvasively, as previously described [44]. The subjects were instructed to fast for at least 12 hours before testing and to abstain from smoking and ingesting alcohol, caffeine or antioxidant vitamins prior to testing. We obtained ultrasound measurements according to the guidelines for ultrasound assessment of the FMD of the brachial artery. Using a 10-MHz linear array transducer probe, the longitudinal image of the right brachial artery was recorded at baseline and then continuously from 30 seconds before to at least two minutes after the cuff deflation that followed suprasystolic compression (50 mmHg above systolic blood pressure (SBP)) of the right forearm for five minutes. The diastolic diameter of the brachial artery was determined semi-automatically using an instrument equipped with a software program for monitoring the brachial artery diameter (Unex Co. Ltd., Nagoya, Japan). The FMD was estimated as the percent change in the diameter over the baseline value at maximal dilation during reactive hyperemia. A total of 10 minutes were allowed to elapse for vessel recovery, after which a further resting scan was taken. Then, 0.3 mg of nitroglycerin was administered, and a final scan was performed five minutes later. We defined patients having endothelial dysfunction as those with FMD<6.0% in the current study based on previous reports [44], [67], [68].

#### Measurement of intima-media thickness (IMT)

Ultrasonography of the carotid artery was performed using a high resolution real-time scanner with a 7.5 MHz transducer, as previously described [40]. The examination was performed with the subject in the supine position, and the carotid bifurcation, as well as the common carotid artery, were scanned on both sides. The maximum IMT value was measured as follows. The carotid artery was scanned in the longitudinal and transverse directions. The site of the most advanced atherosclerotic lesion that showed the greatest distance between the lumen-intima interface and the media-adventitia interface was located in both the right and left carotid arteries. When plaque was detected on ultrasonography, it was observed as localized thickening rather than a circumferential change in the vessel wall. The greatest thickness of the intima-media complex (including plaque) was used for the maximum IMT value. We identified patients having atherosclerosis based on atheromatous plaques of focal increases in IMT≥1.1 mm in accordance with a prior study that showed the normal limit of IMT to be ≤1.0 mm [69].

#### Measurement of ankle-brachial pulse wave velocity (baPWV)

Pulse wave velocity (PWV) measurements were obtained at the bedside of each subject using a volume plethysmographic apparatus (FORM/ABI; Colin, Komaki, Japan) after the subject had rested in the supine position for at least five minutes, as previously described [40]. This instrument allows simultaneous recording of the baPWV and the brachial and ankle BPs on both sides, in addition to recording an electrocardiogram and heart sounds. We defined patients having arterial stiffness as those with baPWV≥1400 since a baPWV≥1400 cm/sec is an independent variable of the risk stratification according to the Framingham score and for the discrimination of patients with atherosclerotic cardiovascular disease [70].

#### Measurement and calculation of the aortic calcification index (ACI)

The ACI was determined as previously described [42], [43]. A non-contrast CT scan of the abdominal aorta was performed. Calcification of the abdominal aorta above the bifurcation of the common iliac arteries was evaluated semi-quantitatively in 10 CT slices at 1 cm intervals. Calcification was considered to be present if an area ≥1 mm^2^ displayed a density ≥130 Hounsfield units. The cross-section of the abdominal aorta on each slice was divided into 12 segments radially. A segment containing an aortic wall with calcification in any section was defined as having aortic calcification. The number of calcified segments was counted in each slice and divided by 12. The values thus obtained for the 10 slices were added together, divided by 10 (the number of slices inspected) and then multiplied by 100 to express the result as a percentage: ACI (%) = (total score for calcification in all slices)/(12 [number of segments in each slice]×10 [number of slices])×100. The ACI was used as a marker for the extent of aortic calcification. We defined CKD patients having abdominal calcification as those with ACI>0%, as described previously [42], [43].

### Statistical analysis

Non-normally distributed variables were expressed as the median (interquartile range) and normally distributed variables were expressed as the mean ± SD as appropriate. A value of P<0.05 was considered to be statistically significant. Differences between groups were analyzed using Student's *t*-test and the Mann-Whitney U-test as appropriate. The Spearman rank correlation was used to determine the correlations between two variables. A multiple logistic regression analysis was applied to test the independent links between the vascular function and potential functional correlates of the outcome variables [71], [72]. A multivariable logistic regression analysis was performed to determine the predictors of baPWV. This multivariate model was built using pre-specified variables including age, gender, MBP, diabetes mellitus, dyslipidemia, eGFR, albuminuria, phosphate, PTH, 1,25D, FGF23 and serum Klotho. The P values, odds ratios (ORs) and corresponding two-sided 95% confidence intervals (CIs) for the predictors are presented. The statistical analyses were performed using the JMP software package release 8 (SAS Institute Inc., Cary, NC, USA).

## Supporting Information

Figure S1 **Box and line plots showing the levels of serum Klotho (pg/mL) according to the estimated glomerular filtration rate (eGFR) (mL/min/1.73 m^2^) or the levels of serum log intact fibroblast growth factor 23 (FGF23) (pg/mL) according to the estimated glomerular filtration rate (eGFR) (mL/min/1.73 m^2^).** The serum soluble Klotho levels significantly decreased in association with declines in eGFR (**A**), while the log-transformed intact FGF23 levels significantly increased in association with declines in eGFR (**B**). (**A**) serum Klotho levels, eGFR≥90 (stage 1), 799.0 (670.6–940.9); eGFR 60–89 (stage 2), 637.4 (546.2–637.4); eGFR 30–59 (stage 3), 595.4 (498.8–773.9); eGFR 15–29 (stage 4), 578.3 (425.9–751.0); eGFR 0–14 (stage 5), 525.1 (389.0–661.4) pg/mL. (**A, B**) eGFR≥90, n = 11; 60–89, n = 36; 30–59, n = 31; 15–29, n = 16, 0–14, n = 20. *, **, *** and **** indicate p<0.05, p<0.01, p<0.005 and p<0.001, respectively. The boxes denote the medians and 25th and 75th percentiles. The lines mark the 5th and 95th percentiles.
(TIF)

Figure S2 **Correlation between the serum Klotho levels (pg/mL) and the other markers of chronic kidney disease-mineral and bone disorder (CKD-MBD).** They include calcium (mg/dL) (A), phosphate (mg/dL) (B), 25-hydroxyvitamin D (25D) (C) and log intact fibroblast growth factor 23 (FGF23) (D) and various markers of vascular dysfunction, including flow-mediated dilatation (FMD) (%) (E), ankle-brachial pulse wave velocity (baPWV) (cm/sec) (F), maximum intima-media thickness (max IMT) (mm) (G) and the aortic calcification index (ACI) (%) (H). The serum Klotho levels tended to be positively correlated with calcium and phosphate and negatively correlated with log intact FGF23, while no significant association was observed between the serum Klotho levels and 25D (**A–D**). Regarding markers of vascular dysfunction, the serum Klotho levels were positively correlated with FMD and negatively correlated with baPWV and max IMT, while the correlation between the serum Klotho levels and ACI was not significant (**E–H**). (**A, B, D, E–H**) N = 114. (**C**) N = 58.
(TIF)

Figure S3 **Multivariate odds ratio for flow-mediated dilatation (FMD) among patients with CKD displayed as the odds ratio (OR) (solid boxes) with 95% confidence intervals (CIs) (horizontal limit lines).** For continuous variables, the unit of change is given in parenthesis based on the multivariate model described in Table S1. MBP, mean blood pressure; eGFR, estimated glomerular filtration rate; PTH, parathyroid hormone; 1,25D, 1,25-dihydroxyvitamin D; FGF23, fibroblast growth factor 23.
(TIF)

Figure S4 **Multivariate odds ratio for maximum intima-media thickness (max IMT) among patients with CKD, displayed as odds ratio (OR) (solid boxes) with 95% confidence intervals (CIs) (horizontal limit lines).** For continuous variables, unit of change is given in parenthesis based on the multivariate model described in Table S2. MBP, mean blood pressure; eGFR, estimated glomerular filtration rate; PTH, parathyroid hormone; 1,25D, 1,25-dihydroxyvitamin D; FGF23, fibroblast growth factor 23.
(TIF)

Figure S5 **Multivariate odds ratio for aortic calcification index (ACI) among patients with CKD displayed as the odds ratio (OR) (solid boxes) with 95% confidence intervals (CIs) (horizontal limit lines).** For continuous variables, the unit of change is given in parenthesis based on the multivariate model described in Table S3. MBP, mean blood pressure; eGFR, estimated glomerular filtration rate; PTH, parathyroid hormone; 1,25D, 1,25-dihydroxyvitamin D; FGF23, fibroblast growth factor 23.
(TIF)

Table S1 **A multiple logistic regression analysis of predictors of FMD≥6.0%.**
(DOC)

Table S2 **A multiple logistic regression analysis of predictors of max IMT≥1.1 mm.**
(DOC)

Table S3 **A multiple logistic regression analysis of predictors of ACI>0%.**
(DOC)
