# Supplementary figures and images for: A Decreased Level of Serum Soluble Klotho Is an Independent Biomarker Associated with Arterial Stiffness in Patients with Chronic Kidney Disease
Source: PLoS One. 2013 Feb 19;8(2):e56695. doi: 10.1371/journal.pone.0056695 (PMC3576368; doi:10.1371/journal.pone.0056695)

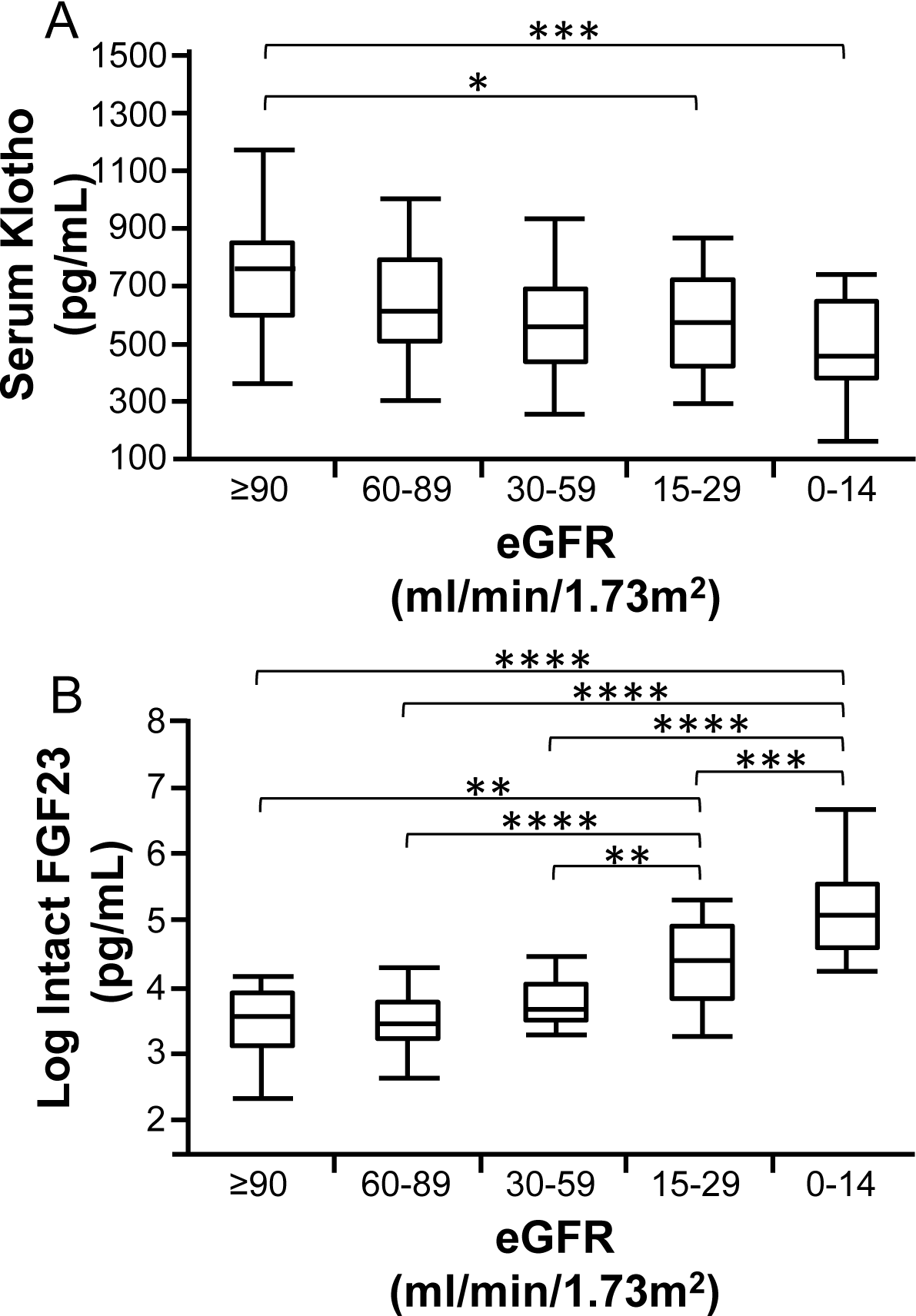

Supplement: Figure S1 — Box and line plots showing the levels of serum Klotho (pg/mL) according to the estimated glomerular filtration rate (eGFR) (mL/min/1.73 m2) or the levels of serum log intact fibroblast growth factor 23 (FGF23) (pg/mL) according to the estimated glomerular filtration rate (eGFR) (mL/min/1.73 m2). The serum soluble Klotho levels significantly decreased in association with declines in eGFR (A), while the log-transformed intact FGF23 levels significantly increased in association with declines in eGFR (B). (A) serum Klotho levels, eGFR≥90 (stage 1), 799.0 (670.6–940.9); eGFR 60–89 (stage 2), 637.4 (546.2–637.4); eGFR 30–59 (stage 3), 595.4 (498.8–773.9); eGFR 15–29 (stage 4), 578.3 (425.9–751.0); eGFR 0–14 (stage 5), 525.1 (389.0–661.4) pg/mL. (A, B) eGFR≥90, n = 11; 60–89, n = 36; 30–59, n = 31; 15–29, n = 16, 0–14, n = 20. *, **, *** and **** indicate p<0.05, p<0.01, p<0.005 and p<0.001, respectively. The boxes denote the medians and 25th and 75th percentiles. The lines mark the 5th and 95th percentiles. (TIF) [file pone.0056695.s001.tif]

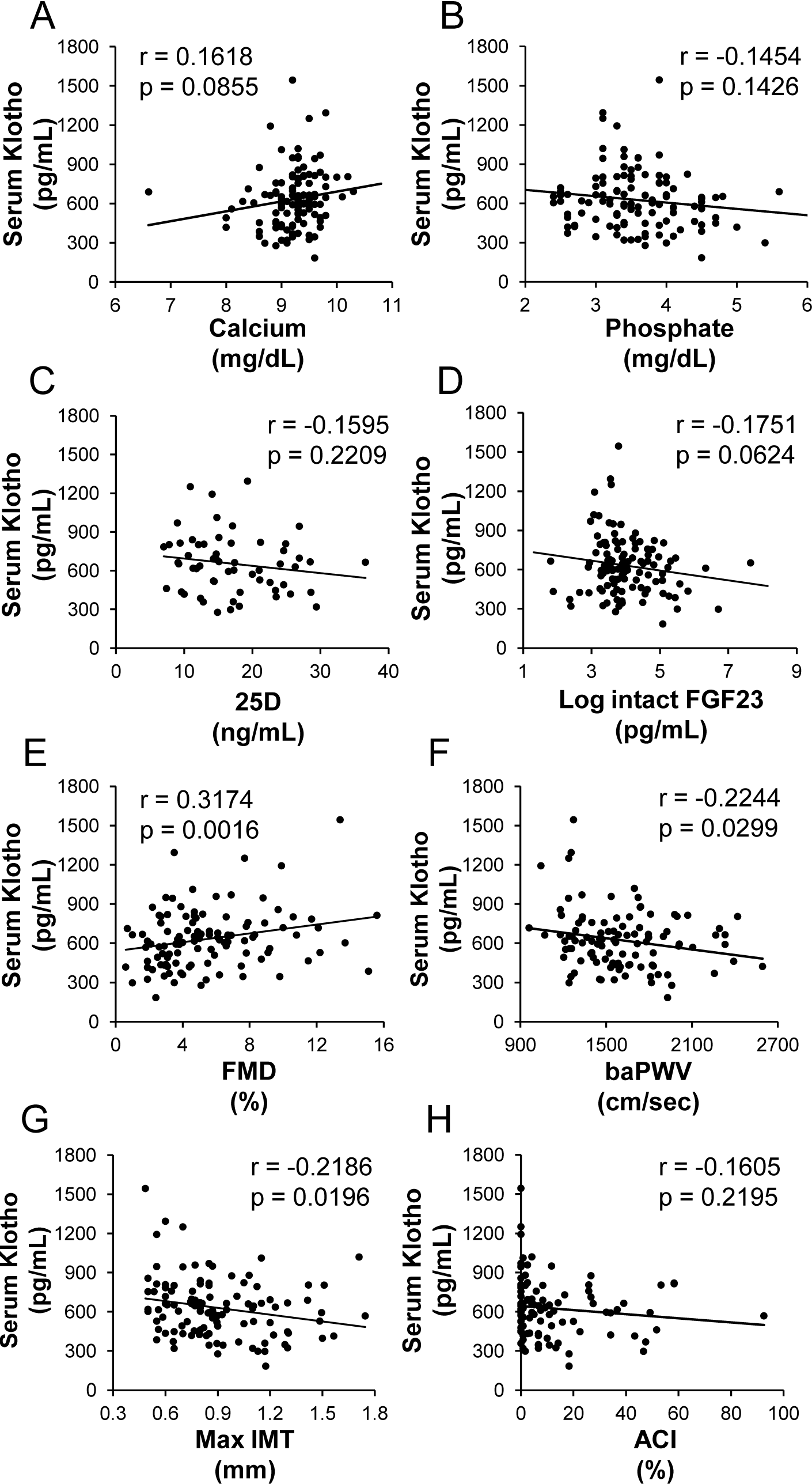

Supplement: Figure S2 — Correlation between the serum Klotho levels (pg/mL) and the other markers of chronic kidney disease-mineral and bone disorder (CKD-MBD). They include calcium (mg/dL) (A), phosphate (mg/dL) (B), 25-hydroxyvitamin D (25D) (C) and log intact fibroblast growth factor 23 (FGF23) (D) and various markers of vascular dysfunction, including flow-mediated dilatation (FMD) (%) (E), ankle-brachial pulse wave velocity (baPWV) (cm/sec) (F), maximum intima-media thickness (max IMT) (mm) (G) and the aortic calcification index (ACI) (%) (H). The serum Klotho levels tended to be positively correlated with calcium and phosphate and negatively correlated with log intact FGF23, while no significant association was observed between the serum Klotho levels and 25D (A–D). Regarding markers of vascular dysfunction, the serum Klotho levels were positively correlated with FMD and negatively correlated with baPWV and max IMT, while the correlation between the serum Klotho levels and ACI was not significant (E–H). (A, B, D, E–H) N = 114. (C) N = 58. (TIF) [file pone.0056695.s002.tif]

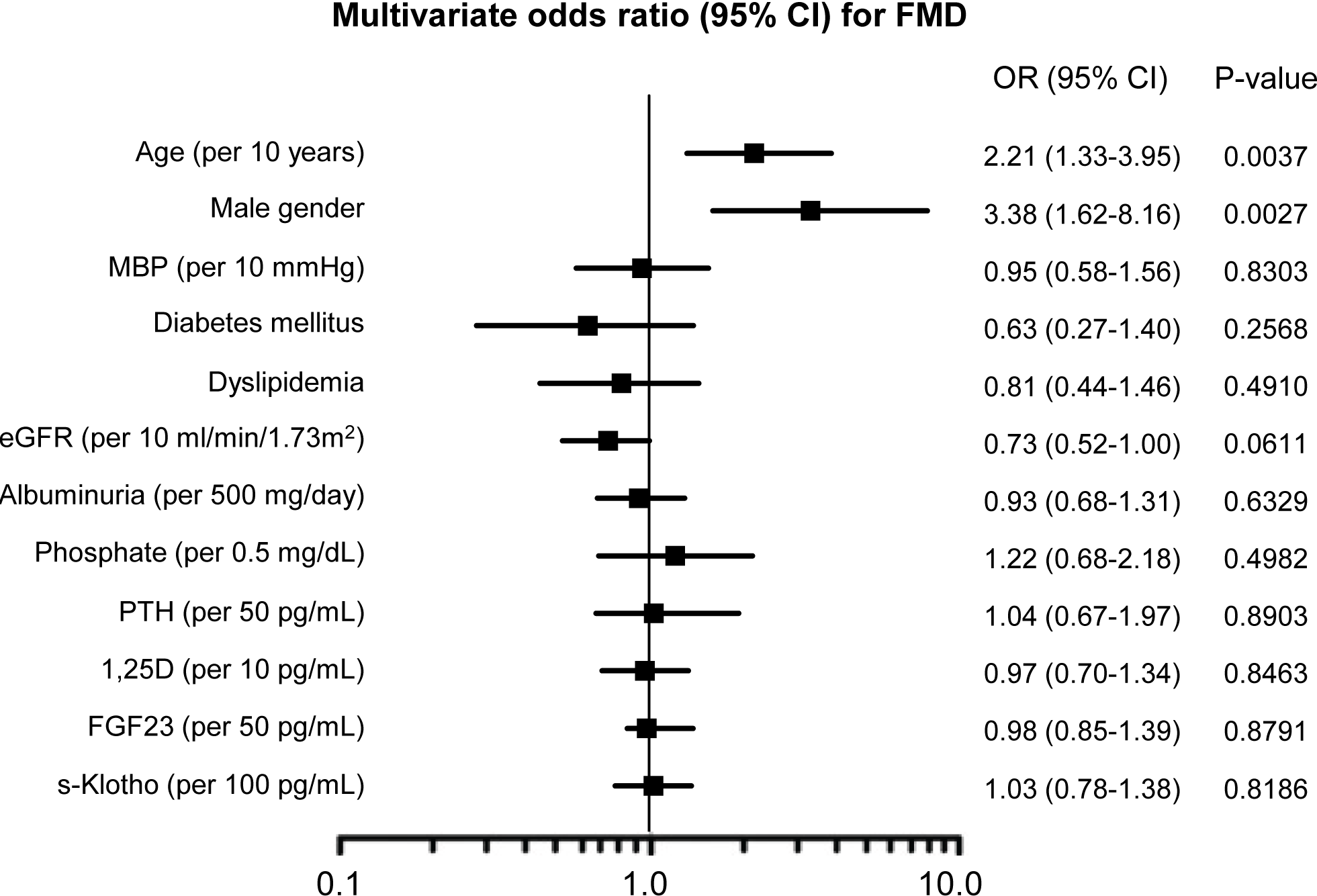

Supplement: Figure S3 — Multivariate odds ratio for flow-mediated dilatation (FMD) among patients with CKD displayed as the odds ratio (OR) (solid boxes) with 95% confidence intervals (CIs) (horizontal limit lines). For continuous variables, the unit of change is given in parenthesis based on the multivariate model described in Table S1. MBP, mean blood pressure; eGFR, estimated glomerular filtration rate; PTH, parathyroid hormone; 1,25D, 1,25-dihydroxyvitamin D; FGF23, fibroblast growth factor 23. (TIF) [file pone.0056695.s003.tif]

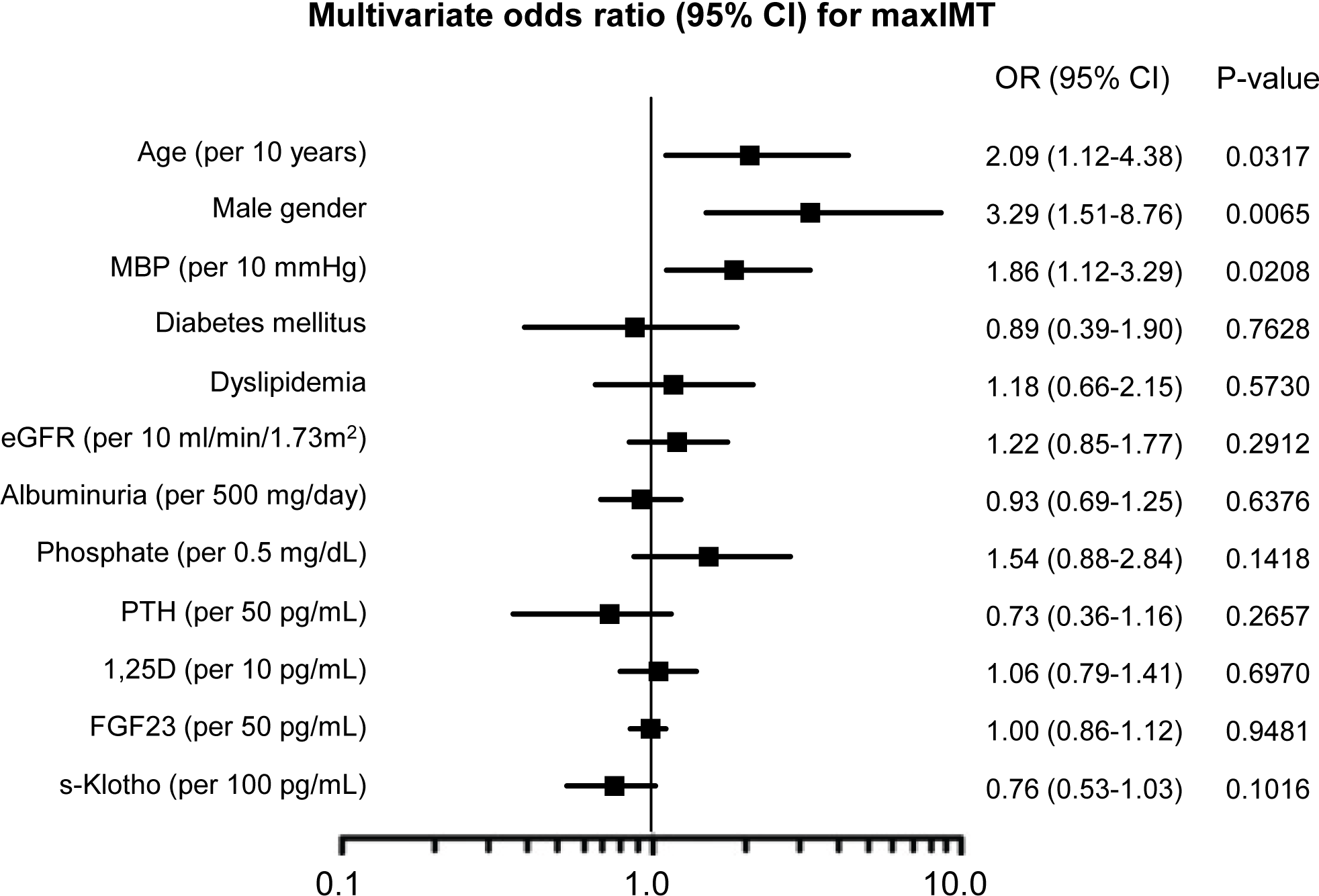

Supplement: Figure S4 — Multivariate odds ratio for maximum intima-media thickness (max IMT) among patients with CKD, displayed as odds ratio (OR) (solid boxes) with 95% confidence intervals (CIs) (horizontal limit lines). For continuous variables, unit of change is given in parenthesis based on the multivariate model described in Table S2. MBP, mean blood pressure; eGFR, estimated glomerular filtration rate; PTH, parathyroid hormone; 1,25D, 1,25-dihydroxyvitamin D; FGF23, fibroblast growth factor 23. (TIF) [file pone.0056695.s004.tif]

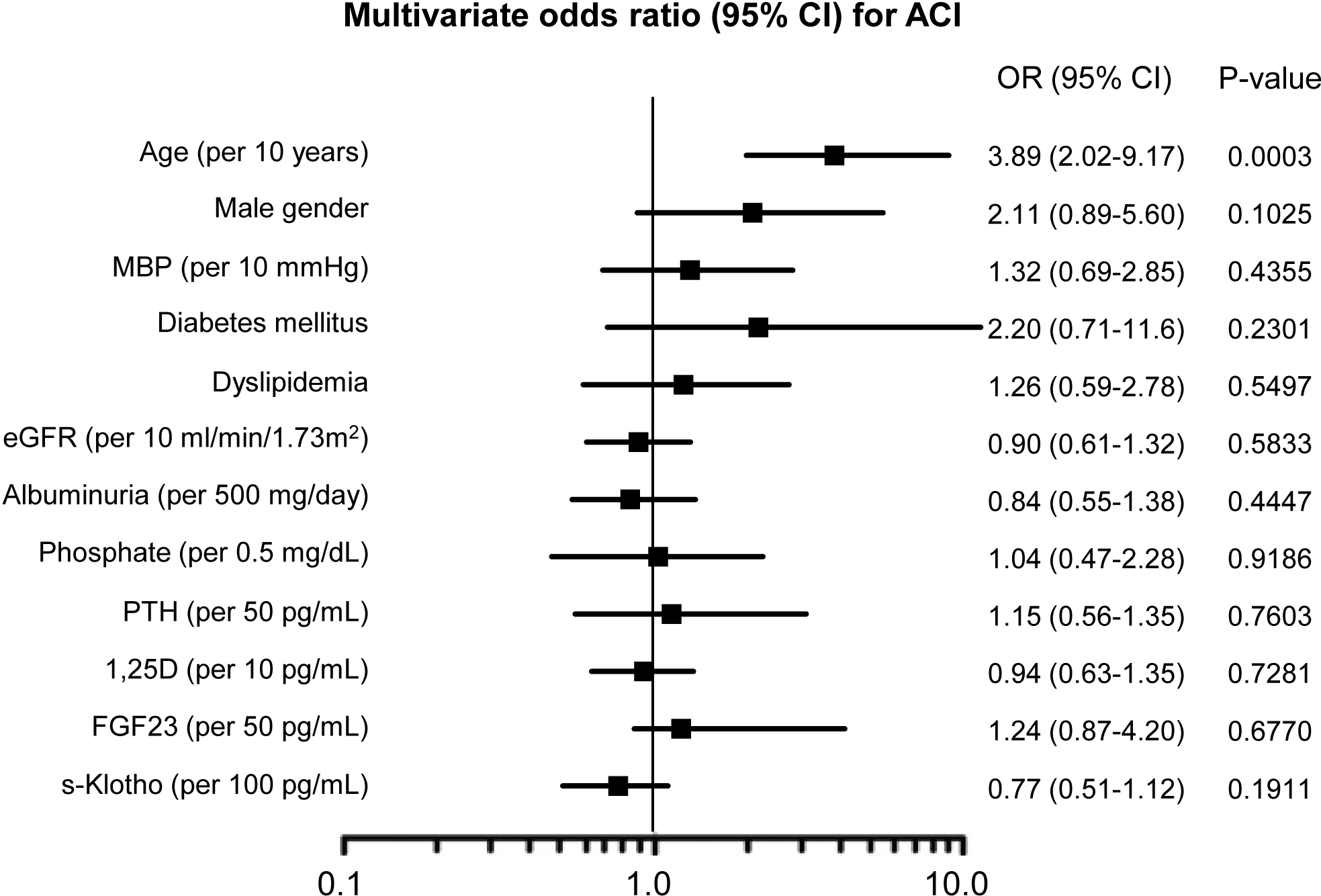

Supplement: Figure S5 — Multivariate odds ratio for aortic calcification index (ACI) among patients with CKD displayed as the odds ratio (OR) (solid boxes) with 95% confidence intervals (CIs) (horizontal limit lines). For continuous variables, the unit of change is given in parenthesis based on the multivariate model described in Table S3. MBP, mean blood pressure; eGFR, estimated glomerular filtration rate; PTH, parathyroid hormone; 1,25D, 1,25-dihydroxyvitamin D; FGF23, fibroblast growth factor 23. (TIF) [file pone.0056695.s005.tif]
