# Supplementary material for: A Decreased Level of Serum Soluble Klotho Is an Independent Biomarker Associated with Arterial Stiffness in Patients with Chronic Kidney Disease
Source: PLoS One. 2013 Feb 19;8(2):e56695. doi: 10.1371/journal.pone.0056695 (PMC3576368; doi:10.1371/journal.pone.0056695)
Supplement: Table S1 — A multiple logistic regression analysis of predictors of FMD≥6.0%. (DOC) [file pone.0056695.s006.doc]

**Table S1. A multiple logistic regression analysis of predictors of FMD ≥ 6.0 %**

|  | β | p |
| --- | --- | --- |
| Metabolic model |  |  |
| serum Klotho | 0.00002 | 0.9859 |
| non HDL | -0.01025 | 0.2330 |
| antihyperlipidemic drugs | -0.08841 | 0.7801 |
| HbA1c (NGSP) | -0.41931 | 0.3171 |
| antidiabetic drugs | 0.07995 | 0.8716 |
| CKD model |  |  |
| serum Klotho | 0.00144 | 0.3485 |
| eGFR | -0.02471 | 0.1083 |
| albuminuria | -0.00036 | 0.2612 |
| Hemoglobin | -0.27721 | 0.1903 |
| CKD-MBD model |  |  |
| serum Klotho | 0.00005 | 0.9721 |
| serum calcium | -0.94623 | 0.2839 |
| serum phosphate | 0.39518 | 0.4903 |
| intact PTH | 0.00179 | 0.7660 |
| 1,25D | -0.00692 | 0.6257 |
| FGF23 | 0.00089 | 0.6789 |

Adjusted for age, gender, mean blood pressure, antihypertensive drug use, drinking and current smoking. CKD, chronic kidney disease; 1,25D, 1,25-dihydroxyvitamin D; eGFR, estimated glomerular filtration rate; FGF23, fibroblast growth factor 23; HDL, high density lipoprotein; MBD, mineral and bone disorder; NGSP, national glycohemoglobin standardization program.
