# Supplementary material for: A Decreased Level of Serum Soluble Klotho Is an Independent Biomarker Associated with Arterial Stiffness in Patients with Chronic Kidney Disease
Source: PLoS One. 2013 Feb 19;8(2):e56695. doi: 10.1371/journal.pone.0056695 (PMC3576368; doi:10.1371/journal.pone.0056695)
Supplement: Table S2 — A multiple logistic regression analysis of predictors of max IMT≥1.1 mm. (DOC) [file pone.0056695.s007.doc]

**Table S2. A multiple logistic regression analysis of predictors of max IMT ≥ 1.1 mm**

|  | β | p |
| --- | --- | --- |
| Metabolic model |  |  |
| serum Klotho | -0.00173 | 0.2168 |
| non HDL | -0.00039 | 0.9563 |
| antihyperlipidemic drugs | -0.23568 | 0.4731 |
| HbA1c (NGSP) | 0.41733 | 0.2515 |
| antidiabetic drugs | 0.58211 | 0.2261 |
| CKD model |  |  |
| serum Klotho | -0.00282 | 0.0722 |
| eGFR | 0.01724 | 0.3534 |
| albuminuria | -0.00003 | 0.9028 |
| Hemoglobin | 0.18885 | 0.3564 |
| CKD-MBD model |  |  |
| serum Klotho | -0.00218 | 0.1611 |
| serum calcium | -0.10384 | 0.2424 |
| serum phosphate | 0.83523 | 0.1321 |
| intact PTH | -0.00554 | 0.2270 |
| 1,25D | 0.02114 | 0.0921 |
| FGF23 | 0.00016 | 0.8933 |

Adjusted for age, gender, mean blood pressure, antihypertensive drug use, drinking and current smoking. CKD, chronic kidney disease; 1,25D, 1,25-dihydroxyvitamin D; eGFR, estimated glomerular filtration rate; FGF23, fibroblast growth factor 23; HDL, high density lipoprotein; MBD, mineral and bone disorder; NGSP, national glycohemoglobin standardization program.
