# Supplementary material for: A Decreased Level of Serum Soluble Klotho Is an Independent Biomarker Associated with Arterial Stiffness in Patients with Chronic Kidney Disease
Source: PLoS One. 2013 Feb 19;8(2):e56695. doi: 10.1371/journal.pone.0056695 (PMC3576368; doi:10.1371/journal.pone.0056695)
Supplement: Table S3 — A multiple logistic regression analysis of predictors of ACI>0%. (DOC) [file pone.0056695.s008.doc]

**Table S3. A multiple logistic regression analysis of predictors of ACI > 0%**

|  | β | p |
| --- | --- | --- |
| Metabolic model |  |  |
| serum Klotho | -0.00265 | 0.1398 |
| non HDL | 0.01309 | 0.2719 |
| antihyperlipidemic drugs | 0.18083 | 0.6963 |
| HbA1c (NGSP) | -0.79309 | 0.4377 |
| antidiabetic drugs | -5.68172 | 0.9079 |
| CKD model |  |  |
| serum Klotho | -0.00237 | 0.2113 |
| eGFR | -0.00054 | 0.9763 |
| albuminuria | -0.00024 | 0.6027 |
| Hemoglobin | -0.29124 | 0.2669 |
| CKD-MBD model |  |  |
| serum Klotho | -0.00226 | 0.2512 |
| serum calcium | 0.22940 | 0.8463 |
| serum phosphate | -0.21838 | 0.7841 |
| intact PTH | 0.00312 | 0.7488 |
| 1,25D | -0.01491 | 0.4500 |
| FGF23 | 0.00945 | 0.4459 |

Adjusted for age, gender, mean blood pressure, antihypertensive drug use, drinking and current smoking. CKD, chronic kidney disease; 1,25D, 1,25-dihydroxyvitamin D; eGFR, estimated glomerular filtration rate; FGF23, fibroblast growth factor 23; HDL, high density lipoprotein; MBD, mineral and bone disorder; NGSP, national glycohemoglobin standardization program.
